# His Majesty the Pig

**Published:** 1918-03-16

**Authors:** 


### HIS MAJESTY THE PIG.

### His Paramount Importance in the Great War.

The importance of the pig as rent-payer has long been appreciated in Ireland; its importance as a fat-producer is very imperfectly appreciated both by the Food Controller and by the committee of experts appointed by the Royal Society. Everyone, even that convenient fiction the man in the street, now knows that the three main ingredients in food are protein, fat, and carbohydrates. As long as the potato crops do not fail, we have a sufficiency of carbohydrates. Our ration of meat, the principal source of protein in our food, is sufficient, though not more than sufficient. Still, when it is supplemented by the protein of which cereals contain some, and peas, beans, and lentils a good deal, the ration of meat is sufficient. But the ration of fat is not sufficient. Weight for weight, man requires as much fat as meat in his diet, and besides its dietetic value, fat is required in many modes of cooking by which the other ingredients in the diet are made palatable, and the necessary variety is introduced into our dishes. Lord Rhondda is a very able man and a most successful coal-owner, and no doubt it was because of his success in administering coal-mines, and of his knowledge of coal, that he was appointed to the position of Food Controller, for everyone knows what an immense quantity of coal is eaten by the inhabitants of these islands; but it is doubtful whether Lord Rhondda knows that fat is an ingredient in pastry, that it is necessary for frying, or that it enters more or less into almost every operation of the cuisine; and if Lord Rhondda does not know we may be sure that the learned and eminent physiologists on the committee appointed by the Royal Society do not know. However, so it is; and fat has a double importance in our diet, first as itself a necessary ingredient in food, and second as a necessary medium for making other foods palatable, and for varying the monotony of the ways in which our food is presented to us.

The other ingredients in our diet are, if not superabundant, yet sufficient; but we are short of fat, and in these circumstances it would seem only common sense to cultivate and encourage the readiest means by which fat can be produced, which is unquestionably and beyond all comparison the breeding of pigs. Few of the vegetable crops grown in this country produce much fat. We grow no flax for seed, and if we did, linseed oil is not a customary article of diet, and is perhaps not a very suitable one. We grow neither the cocoanut nor the olive. We do not grow even the castor-oil bean in commercial quantity, and even if we devote our glass-houses to the purpose, the fat thence obtained would scarcely be suitable for cooking. Cotton-seed oil is wholesome, and, in fact, we consume a great deal of it in the fond imagination that it is olive oil; but again, we do not grow cotton in this country, for our climate is not suitable for it. We might grow rape-seed, but we should have to be educated into adopting rape-seed oil as an article of diet. But we need no education to induce us to eat bacon or pork, or to use lard in our culinary operations; and all vegetable fats are subject to the drawback that they are produced only once a year, and that they need for their extraction manufacturing processes, sometimes of considerable complexity. There is a certain amount of fat to be obtained from fish, and from the birds that feed on fish, but, as we see in the case of cod-liver oil, the fat is not always very palatable, and is, in fact, never very delicious. Fat is to be obtained from whales also, but it must be fetched from afar; its quantity is not great, and its quality is to seek; and no doubt there are other sources of fat in the animal and vegetable kingdoms. Even minerals yield their quota of fat, and it is conceivable that we might utilise for food or for cooking the heavy mineral oils that are now used for lubricating machinery; but the prospect is not attractive, nor are the mineral fats of high nutritive value.

The fat of the pig is free from all these defects. The pig is easily cultivated in this country, as long experience shows. Its fat is very palatable, and, as already mentioned, we need no education to induce us to adopt it. There is no prejudice to overcome. It is produced all the year round, and its quantity is limited only by our own wills in restricting the breeding of pigs. Fat is produced more quickly by breeding pigs than by any other known means. It does not need to be imported, but can be grown in this country to any extent. Why, then, does the Food Controller, at the instance of the Committee of the Royal Society, discourage the breeding of pigs as a source of fat?

Because, say they, it is wasteful. It requires 7 lb. of barley-meal to make 1 lb. of fat, and in making the 1 lb. of fat we are wasting 6 lb. of material. Let us eat the barley-meal ourselves and not give it to those wasteful pigs. But, as a matter of fact, we don't eat barley-meal, and if we did, we should be eating barley-meal and not fat, and it is fat that we want. We cannot fry pancakes in barley-meal, or make fritters with it, or smear the insides of our dishes, or use it to lighten our pastry, or spread it on our bread in lieu of butter; and, moreover, though barley-meal is the conventional food for pigs, and they thrive upon it, and produce fat rapidly from it, yet it is not the only food out of which pigs can make fat. Pigs naturally run to fat, and can produce fat out of almost any food they eat; and they eat many things that we do not eat, many things that we should not like to eat, many things that are not, as barley-meal is, clean and sweet and fit for consumption by man. Out of these things they make fat, and make it more quickly than it can be made by any other process; and we want fat—we want it badly, and we want it at once. Therefore, in spite of the Royal Society and Lord Rhondda, we say to our readers, Keep pigs. Keep as many pigs as you can feed, and, above all, do it now!

